# New highlights on effects of rapid palatal expansion on the skull base: a finite element analysis study

**DOI:** 10.1590/2177-6709.26.6.e2120162.oar

**Published:** 2021-12-15

**Authors:** Manuel Gustavo Chávez SEVILLANO, Daniel Takanori KEMMOKU, Pedro Yoshito NORITOMI, Luciana Quintanilha Pires FERNANDES, Jonas CAPELLI, Cátia QUINTÃO

**Affiliations:** 1 Universidad Nacional Mayor de San Marcos, Facultad de Odontología, Departamento de Estomatologia Pediatrica (Lima, Peru).; 2 Universidade do Estado do Rio de Janeiro, Departamento de Ortodontia (Rio de Janeiro/RJ, Brazil).; 3 Centro de Tecnologia da Informação Renato Archer, Núcleo de Tecnologias Tridimensionais (Campinas/SP, Brazil).

**Keywords:** Finite element analysis, Palatal expansion technique, Skull

## Abstract

**Objective:**

The objective of this study was to evaluate the effect of the rapid palatal expansion (RPE) on the pterygoid process (PP), spheno-occipital synchondrosis (SOS) and sella turcica (ST) in the skull of a patient with transversal maxillary collapse, and identify the distribution of mechanical stresses and displacement, by finite element analysis (FEA).

**Methods:**

Cone-beam computed tomography (CBCT) was employed to examine the skull of a patient in this study. The patient was a 13-year-old boy, with Class II skeletal relationship due to transverse atresia and maxillary protrusion. The computer-aided design (CAD) geometry of skull was imported into the SimLab v. 13.1 software, to build the finite element mesh. For the simulation, a displacement of 1 mm, 3 mm and 5 mm in a transverse direction was defined at the midpalatal suture, thereby representing the RPE. For the analysis of results, maximum principal stress (MPS) and displacements were evaluated by identifying different nodes, which were represented by the points as per the areas of interest in the study.

**Results:**

In MPS, the maximum tensile stress was found at point 2 (366.50 MPa) and point 3 (271.50 Mpa). The maximum compressive stress was found at point 8 (-5.84 Mpa). The higher displacements in the transversal plane and the lateral segment were located at point 1 (2.212 mm), point 2 (0.903 mm) and point 3 (0.238 mm).

**Conclusions:**

RPE has a direct effect on PP, SOS and ST in the Class II model skeletal relationship with a transversal maxillary collapse. PP supported a higher tensile stress and displacement.

## RESUMO

**Objetivo:**

O objetivo do presente estudo foi avaliar o efeito da expansão rápida da maxila (ERM) no processo pterigoide (PP), sincondrose esfeno-occipital (SEO) e sela túrcica (ST) no crânio de um paciente com atresia maxilar, e identificar a distribuição de tensões mecânicas e deslocamento, por meio do método dos elementos finitos (MEF).

**Métodos:**

Foi utilizada uma tomografia computadorizada de feixe cônico (TCFC) para registrar o crânio de um paciente do sexo masculino, de 13 anos de idade, com relação esquelética de Classe II por protrusão da maxila e atresia maxilar. A geometria CAD (desenho assistido por computador) do crânio foi importada para o *software* SimLab 13.1 para construir a malha de elementos finitos. Para simulação, foram definidos deslocamentos, em direção transversal, de 1 mm, 3 mm e 5 mm a partir da sutura palatina mediana, representando, assim, a ERM. Para a análise dos resultados, a tensão máxima principal (TMP) e os deslocamentos foram avaliados por meio da identificação de diferentes nós, que foram representados pelos pontos de acordo com as áreas de interesse do estudo.

**Resultados:**

Na TMP, a tensão máxima de tração foi encontrada nos pontos 2 (366,50 MPa) e 3 (271,50 MPa). A tensão máxima de compressão foi encontrada no ponto 8 (-5,84 MPa). Os maiores deslocamentos no plano transversal e no segmento lateral foram localizados nos pontos 1 (2,212 mm), 2 (0,903 mm) e 3 (0,238 mm).

**Conclusões:**

A ERM teve efeito direto sobre PP, SEO e ST no modelo de relação esquelética de Classe II com atresia maxilar. O PP demonstrou uma maior tensão de tração e deslocamento.

## INTRODUCTION

Posterior dental crossbites and transversal maxillary collapse are often treated with rapid palatal expansion (RPE), which involves increasing the perimeter of the dental and skeletal arches and skeletal Class III treatment combined with maxillary protraction.^1^ ^,^ ^2^ The main anatomical objective is to open the midpalatal suture (MPTS), which becomes harder with the age of an individual. However, there is not an absolute correlation between the ossification and the biological age.^3^

In majority of adult patients with transversal maxillary collapse, for whom surgery does not remains a viable option, RPE protocols were proposed that involved more tooth than skeletal movement.^4^ ^,^ ^5^ Through the application of bone anchorage via micro-implant assisted rapid palatal expansion (MARPE), the opening of the MPTS was made possible in adult patients.^6^ ^,^ ^7^ The stress that can be withstand by the craniofacial structures during the RPE has been registered for both conventional approach^8^ ^,^ ^9^ and MARPE.^10^

Due to bone elasticity,^11^ the stress generated on the craniofacial structures during RPE are correlated with age and the ossification degree from the bone sutures, especially in adult patients. Many sutures around maxillary bones are opened during RPE, but pterygopalatine suture was found to be among the ones that offered a greater resistance to MPTS.^12^ However, partial success has been achieved when the sutures were even opened with the MARPE.^13^

There is an important anatomical relationship between the maxillary region of the skull base and the pterygoid processes (PP) of the sphenoid bone. It happens because RPE also affects the deep regions and the neurocranium and viscerocranium. The transmission of mechanical stress produced by the expander appliance during the opening of the MPTS may affect the anatomical structures directly or indirectly,^14^ for example in the case of the spheno-occipital synchondrosis (SOS),^12^ ^,^ ^15^ ^,^ ^16^ PP,^8^ ^,^ ^9^ ^,^ ^14^ sella turcica (ST)^17^ and some cranial base foramina with its vascular and nerve content.^17^ ^,^ ^18^ Although there was described in the literature that human bone has the ability to adapt to the application of mechanical loads, these must be functional and cyclical,^19^ which is not achieved during RPE.

The finite element analysis (FEA) allows the simulation of the system of mechanical forces that act on the human skull during the process of a conventional RPE or MARPE, and analyses the response of such mechanical loads on the neurocranium and viscerocranium.^10^ Knowing that this delicate structure might be affected by heavy loads generated during the process of RPE, the knowledge of these effects presents a great importance for the monitoring and programming of the treatment of transversal maxillary collapse.

The present research evaluated the effect of RPE on PP, SOS and ST in the skull of a patient with transversal maxillary collapse, in order to identify the distribution of mechanical stresses and displacements at the specific points of these anatomical structures, by the FEA method.

## MATERIAL AND METHODS

The ethics committee of Piracicaba Dental School - State University of Campinas approved this study (Protocol number: 056/2013). 

## GEOMETRY AND FINITE ELEMENT MODEL ACQUISITION

**Figure 1:**
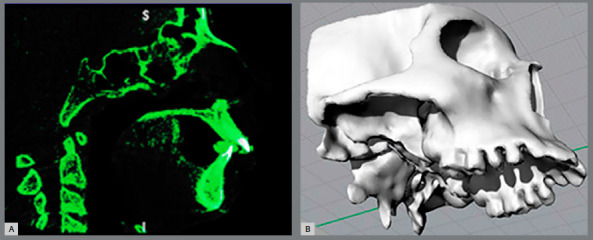
A) Image segmented and (B) CAD geometry in Rhinoceros 5.0 software.

Cone-beam computed tomography (CBCT) was employed to examine the patient’s skull in this study. The case used is of a 13-year-old male with Class II skeletal relationship by maxillary protrusion, transversal maxillary collapse, complete permanent dentition, posterior dental crossbite and non-ossified SOS. The CBCT images, presenting slice thickness at the intervals of 0.25 mm, were imported in the InVesalius 3.0 software (Center for Information Technology “Renato Archer”, Campinas, Brazil) and segmented through the grayscale threshold, to obtain the three-dimensional (3D) surface of maxilla and skull base (Fig 1). The selected bone structure was converted into a 3D stereolithography (STL) surface.

**Figure 2:**
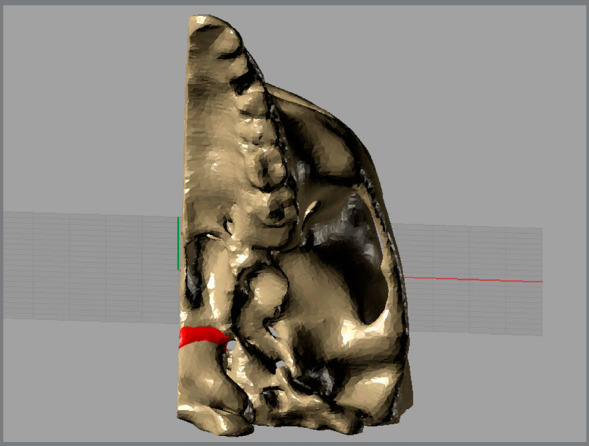
CAD geometry with the solid SOS.

The CAD geometry was constructed in Rhinoceros 5.0 software (McNeel & Associates, Seattle, WA). The modelling was performed through the STL surface conversion into NURBS surfaces. The MPTS and SOS spaces were filled with solids, thereby corresponding to the connective and cartilage tissues, respectively (Fig 2).

In order to build the finite element mesh, the CAD geometry of the skull was imported into the SimLab 13.1 software (Altair Engineering). Tetrahedral elements were used for mesh generation, which resulted in a mesh composed by 344,808 elements and 596,966 nodes. The properties of materials were considered to be linearly elastic and isotropic. The structures were assigned according to the properties of bone, cartilage for SOS and connective tissue for MPTS. The properties of each material defined by Young’s modulus and Poisson’s ratio were used as in previous studies (Table 1).^20^ ^,^ ^21^ ^,^ ^22^ For better observation of the biomechanical effects on the PP, SOS and ST, from the complete skull model a sagittal section was presented through the finite element model.^23^

**Table 1:** Mechanical properties used in previous studies.

Material	Young’s modulus (Mpa)	Poisson’s ratio
Bone^20^	14000 Mpa	0,3
Midpalatal suture^21^	1 Mpa	0,3
Spheno-occipital synchondrosis^22^	24 Mpa	0,3

### BOUNDARY CONDITIONS AND LOADING

The boundary condition was defined by zero displacement and zero rotation on the nodes along with the foramen magnum margin. Also, the shape and loads were made symmetric around the X-axis (transverse). The activation was only performed by forced displacement for the purpose of the maxillary expansion. In the region of the MPTS, a mathematical condition was specified for symmetry and load, which was reproduced symmetrically on the opposite side. A transversal movement of 0.5 mm, 1.5 mm and 2.5 mm per side was simulated in the model. Due to the symmetry condition, this simulation was equivalent to an activation of 1 mm, 3 mm and 5 mm, respectively. 

### STRESS ANALYSIS

**Figure 3:**
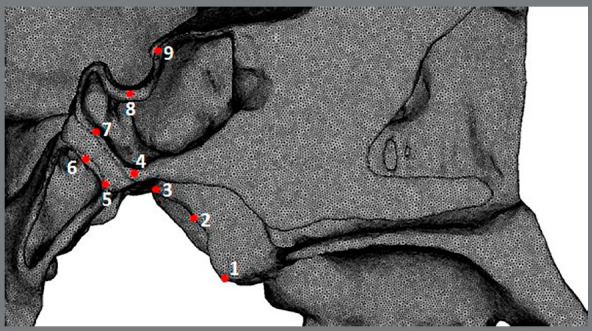
Points for stress evaluations in the sagittal view.

Using the SimLab v. 13.1 software, the displacement and maximum principal stress (MPS) were evaluated by identifying the different nodes, which were represented by points used in previous studies (Fig 3 and Table 2).^24^

**Table 2:** Points and anatomical structures.

Point	Selected nodes on
1	The most inferior and posterior point of the medial pterygoid plate.
2	The most posterior and middle point of the medial pterygoid plate.
3	The most upper and posterior point of the medial pterygoid plate.
4	The most inferior point of the posterior surface of the sphenoid body.
5	The most inferior point of the anterior surface of the basilar part of the occipital bone.
6	The point of intersection between the S-Ba line and the anterior surface of the basilar of the occipital bone.
7	The point of intersection between the S-Ba line and the posterior surface of the sphenoid body.
8	The deepest point of the floor of the sella turcica.
9	The uppermost point of the tuberculum sellae.

## RESULTS

### MAXIMUM PRINCIPAL STRESS (MPS)

The analysis of MPS presented the positive values (tensile stress) and negative values (compressive stress). When a 5-mm expansion was produced, the MPS was found at points 2 (366.50 MPa), 3 (271.50 MPa) and 4 (146.50 MPa). When the expansion was limited to 3 mm, the values of high tensile stress were registered on the same points 2, 3 and 4 (219.90 MPa, 162.90 MPa and 88.08 MPa, respectively). 

When the 5-mm expansion was produced, higher tensile stress was found on the previous region of SOS at points 4 (146.80 MPa) and 7 (35.14 MPa), as compared with the posterior region at points 5 (21.41 MPa) and 6 (4.36 MPa). The same tensile stress pattern was observed with the expansions of 1 mm and 3 mm. 

**Figure 4:**
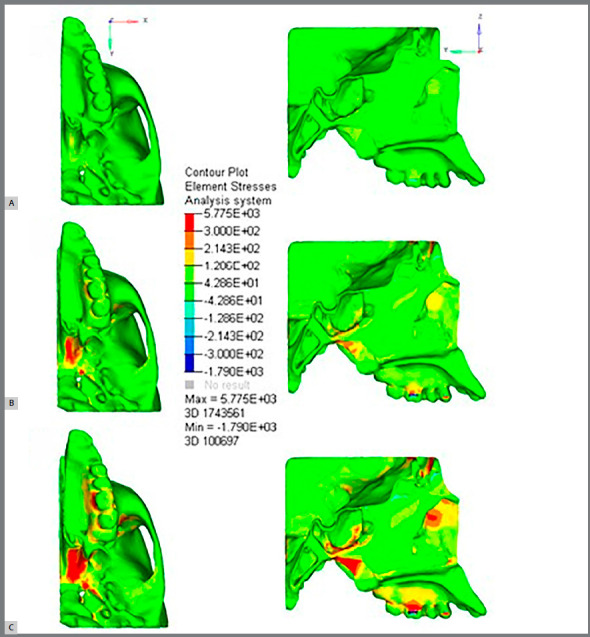
View in the axial and sagittal plane of MPS in megapascals in (**A**) 1mm, (**B**) 3mm and (**C**) 5mm of expansion. The color scale shows the high tensile stress areas in red colors and low tensile stress areas in green. Dark blue color shows the compressive stress areas.

Compressive stress was found in ST for expansion simulations of 1 mm, 3 mm and 5 mm at point 8 with stress of -1.17 MPa, -3.51 MPa and -5.84 MPa, respectively. The tuberculum sellae showed a higher tensile stress at point 9 (39.32 MPa) as compared with the 5-mm expansion (Video 1, Fig 4 and Table 3).

**Video 1:**
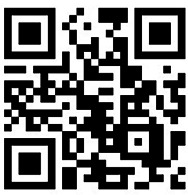
Analysis of maximum principal stress during rapid palatal expansion simulation. The color scale shows the high tensile stress areas in red colors and low tensile stress areas in green. Dark blue color shows the compressive stress areas.

### DISPLACEMENTS

**Figure 5:**
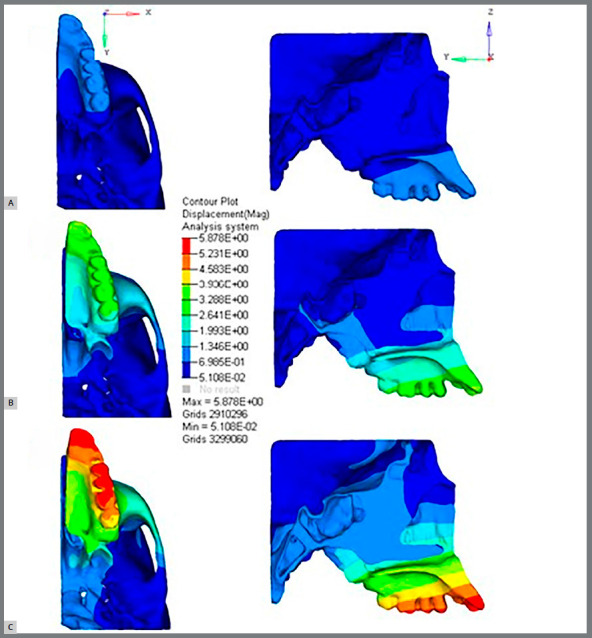
View in the axial and sagittal plane of displacement in (**A**) 1mm, (**B**) 3mm and (**C**) 5mm of expansion. The color scale shows the high displacement areas by red colors and low displacement areas by light blue. A pattern of displacement can be clearly seen in a “V” pattern in the axial view.

When the 5-mm expansion was performed, the higher displacements were registered on the transversal plane (X-axis) at points 1 (2.212 mm), 2 (0.933 mm) and 3 (0.238 mm) with a lateral displacement (Fig 5 and Table 4). The same points were also displaced to the upper and posterior positions, with the displacement being larger in magnitude at point 1 (-1.093 mm and -0.721 mm, respectively) and smaller in magnitude at point 3 (-1 mm and -0.473 mm, respectively). The points located at the anterior area of the SOS (points 4 and 7) and the ones located at the posterior zone (points 5 and 6) registered displacements in the upper and posterior positions, with smaller magnitude observed at the points in the posterior region. The ST region (point 8) and the tuberculum sellae (point 9) were also displaced in the superior (-1.003 mm and -0.884 mm, respectively) and posterior (-0.294 mm and -0.091 mm, respectively) directions. Points 4, 5, 6 and 7 did not showed displacement in the lateral direction (Figure 5, Table 4 and Video 2).

**Video 2:**
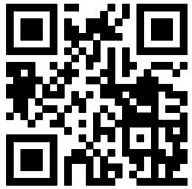
Analysis of displacements during rapid palatal expansion simulation. The color scale shows the high displacement areas in red color, and low displacement areas in light blue. A pattern of displacement can be clearly seen in a “V” pattern in the axial view.

## DISCUSSION

Transverse atresia of the maxilla is mainly corrected by conventional RPE, which involves the application of lateral forces on the palate and the teeth for opening the MPTS.^1^ Also, it has been determined that the “V” pattern that has been opened via MPTS is related to the difficulty of circuit breaker to open the pterygopalatine suture.^12^

The conventional RPE and the MARPE cause the MPTS opening, and transversal forces may be transmitted to the skull base via a connection between the maxilla and PP.^9^ ^,^ ^14^ It significantly prevents the forces to reach the skull base, thereby achieving the opening of the pterygopalatine suture, which would further imply the separation of pyramidal process of the palatine bone from PP.^25^ The opening of pterygopalatine suture in adolescents and young adults is accompanied by fractures due to the strong interdigitation between bone surfaces,^26^ therefore, some studies claim that conventional RPE would not be able to open it.^12^ Using a different type of MARPE, a success rate of 53% was achieved in the opening of the pterygopalatine suture^13^ and 100% of the MPTS. Reaching the opening of the MPTS and not the pterygopalatine suture suggests that strong stress would be transmitted to the base of the skull causing collateral biomechanical effects.^17^ ^,^ ^18^

Wolff^19^ stated that, when human bone is subjected to mechanical loads, the bone structure in its internal and external constitution undergoes a remodeling as a mechanism of adaptation to these forces. A fundamental characteristic of these forces is that they are functional and cyclical,^19^ such as occlusion forces. During the RPE, some intense forces created are neither functional nor cyclical; therefore, we suggest that it is difficult to find a healthy adaptation remodeling in the compromised internal bone structures.

Conventional RPE and MARPE simulation through FEA has been identified as an effective method in the field of orthodontics for the study of the forces produced on the craniofacial structures.^9^ ^,^ ^17^ Since the extent and effects on the skull base should also be known to every orthodontist, the present study simulated the displacement of the palatal process after assuming to overcoming the resistance of the MPTS, circummaxillary sutures, teeth and periodontal ligament with openings of 1 mm, 3 mm and 5 mm, as it was performed in previous studies^8^ and without opening the pterygopalatine suture as in many clinical situations, especially in adults.^13^ The displacement of the palatal process was simulated in almost parallel way, following the clinical responses obtained in a study in CBCTs by Cantarella et al.^13^ Regardless of whether the load force was applied to the teeth or micro-implant, only the opening of the MPTS was simulated as reported protocols.^8^ ^,^ ^27^

In the MPS analysis, tensile stress was found at points 1, 2 and 3 of the medial pterygoid plate, with point 1 showing a lower value. This stress distribution agrees with the findings in the studies of Iseri et al^8^, Baldawa and Bhad.^28^ If the pterygopalatine suture is not open, it is likely that these tensile stresses occur due to lateral bend of PP during the RPE, as described by Jafari et al^9^ and Iseri et al.^8^ In another study that involved the simulation of opening of the pterygopalatine suture, a significant reduction in tensile stress on PP and SOS was observed.^27^

It is important to note that tensile stress decreases at points 3 and 4, as compared with point 2, what could be related to the proximity to the SOS cartilage and its ability to absorb the forces.^29^ This effect can be clearly observed by the difference in the tensile stress between points 4 and 7 (before SOS) and points 5 and 6 (after SOS). Thilander and Ingervall^29^ found collagen fibres in SOS, arranged in the longitudinal direction of the clivus, which could probably mean the preparation for tensile stress distribution. It is possible to suggest that when SOS is fully ossified, the tensile stress on the base of the skull could be much higher. The tensile stresses that SOS receives were also observed in previous studies.^17^ ^,^ ^18^ ^,^ ^27^

Although tensile stress reduces its value at point 3 in relation to point 2, it is one of the highest found in this study, and because of its proximity to the base of the skull, it could be transmitting forces on the foramina of the base of the skull with vascular and nervous content, causing bone responses such as microfractures or bone resorption.^17^ ^,^ ^18^ However, Thilander and Ingervall^29^ found the cartilage regions at the ST of skulls in older patients, which could cause lower values of stress in this region. Not only tensile stress was identified at the base of the skull, but also compressive stress at ST (point 8), which is in agreement with the findings of Holberg et al.^27^ The magnitude of the stress recorded in patients could be related to age, since Holberg^18^, Holberg and Rudzki-Janson^17^ performed comparative simulations by using different values of Young’s modulus and Poisson coefficients to identify the young bone and adult bone. They found greater stress and smaller displacements for the adult bones.

In addition to the body, that was considered elastic and linear, it can be seen in Tables 3 and 4 that point 1 had greater displacement and less tensile stress than points 2 and 3, and point 3 had less displacement and less tensile stress than point 2. We suggest that this result is due to the presence of cartilage and the complex geometry of the anatomical structure.^20^

**Table 3:** MPS values at the anatomical points evaluated.

Point	Maximum Principal Stress (MPa)		
	1 mm	3 mm	5 mm
1	1.00	3.00	5.01
2	73.30	219.90	366.50
3	54.30	162.90	271.50
4	29.36	88.08	146.80
5	4.28	12.85	21.41
6	0.87	2.61	4.36
7	7.03	21.08	35.14
8	-1.17	-3.51	-5.84
9	7.86	23.59	39.32

*Displacement: 1mm, 3mm, and 5mm.

**Table 4:** Displacement from 5mm at the anatomical points evaluated.

Point	Displacement (5mm)		
	X	Y	Z
1	2.212	-0.721	-1.093
2	0.903	-0.604	-1.072
3	0.238	-0.473	-1
4	0	-0.555	-1.055
5	0.01	-0.135	-0.944
6	0	-0.178	-0.919
7	0	-0.469	-1.108
8	0	-0.294	-1.003
9	0	-0.091	-0.884

*Axis: X, Y and Z.

When the 5-mm expansion was performed, the greatest displacements were recorded in the transverse plane in PP at points 1, 2 and 3, which are consistent with the results of previous studies.^8^ ^,^ ^9^ ^,^ ^28^ The same points were also displaced in the superior and posterior directions, which suggest an opening of the MPTS in “V” pattern in the axial and coronal planes. The movements recorded in the points before the SOS (points 4 and 7) and the later ones (points 5 and 6) would be related to SOS movements, as described by other studies.^15^ ^,^ ^16^ ^,^ ^18^ The movements was simulated with the objective of finding significant displacements at the maximum expansion of 5 mm, and at points 8 and 9, the movement was found to be almost negligible. By observing the simulation of the movement of the MPTS in the axial plane, the opening pattern can be observed in all models with the expansions of 1 mm, 3 mm and 5 mm, as described in previous studies.^14^

Knowing that stress difference found between the skull model is based on morphology, because the geometry is fundamental to the mechanical response,^11^ ^,^ ^20^ the present study has certain limitations. In order to be able to simplify and represent the biomechanical procedure, the complete skull model was assumed to be isotropic and linearly elastic.^20^ Since it is important to know the different types of stresses that occur on the craniofacial structures, the MPS analysis was selected instead of the equivalent von-Mises and the minimum principal stress analysis. This also allows a qualitative and quantitative assessment of stress forces. The bone structures were analyzed by MPS to determine the tensile (positive values) and compressive (negative values) stresses. Although the analysis has expressed the MPS stress values, this study was not intended to determine absolute stress magnitudes, but assisted in the localization of high and low stress distribution based on PP, SOS, and ST geometry.^20^

The meshes corresponding to spongy bone tissue, teeth, periodontal ligament and circummaxillary sutures were not individualized since, in this study, the beginning of the expansion simulation starts assuming that the initial resistance of these anatomical structures had already been overcome (after the initial opening of the MPTS), as described in a previous study.^8^ ^,^ ^27^ Also, the application of molar and premolar forces was not simulated, but the opening of the MPTS following the protocols of previous studies.^27^ ^,^ ^30^

The present study did not compare the effects of conventional RPE and MARPE. Since both have as their main objective the opening of the MPTS, the biomechanical effects are highlighted after the MPTS begins to open.^30^ The points used to identify the researched anatomical structures were selected from a previous study, which aimed to examine the biomechanical response of the SOS to RPE.^24^

The FEA is a strong tool to create hypotheses in the field of biomechanics, since the results obtained may differ from the actual clinical results, because a computational model cannot reproduce all the biological variables that occur in the real clinical situations. A single finite element model could not represent each and every clinical situation, so future mechanical tests and the analyses of conventional clinical models are required to corroborate the present results. Therefore, this study does not induce to limit the use of conventional RPE or MARPE, but it seeks to know the biomechanical impact of extreme forces on the delicate craniofacial anatomical structures that could occur in adolescents patients. 

## CONCLUSIONS

1. RPE has a direct effect on PP, SOS and ST in a patient with Class II model skeletal relationship with transversal maxillary collapse.
2. The Class II model undergoing RPE supports a higher tensile stress and displacement at PP.
3. The compressive stress on the ST is relatively lower in Class II model.

## References

[1] Hass AJ (1961). Rapid expansion of maxillary dental arch and nasal cavity by opening the midpalatal suture. Am J Orthod.

[2] Garrett BJ, Caruso JM, Rungcharassaeng K, Farrage JR, Kim JS, Taylor GD (2008). Skeletal effects to the maxilla after rapid maxillary expansion assessed with cone-beam computed tomography. Am J Orthod Dentofacial Orthop.

[3] Angelieri F, Cevidanes LH, Franchi L, Gonçalves JR, Benavides E, McNamara JA (2013). Midpalatal suture maturation classification method for individual assessment before rapid maxillary expansion. Am J Orthod Dentofacial Orthop.

[4] Halicioglu K, Yavuz I, Ceylan I, Erdem A (2014). Effects of face mask treatment with and without rapid maxillary expansion in young adult subjects. Angle Orthod.

[5] Handelman CS, Wang L, BeGole EA, Haas AJ (2000). Nonsurgical rapid maxillary expansion in adults report on 47 cases using the Haas expander. Angle Orthod.

[6] Carlson C, Sung J, McComb RW, Machado AW, Moon W (2016). Microimplant-assisted rapid palatal expansion appliance to orthopedically correct transverse maxillary deficiency in an adult. Am J Orthod Dentofacial Orthop.

[7] Brunetto DP, Sant'Anna EF, Machado AW, Moon W (2017). Non-surgical treatment of transverse deficiency in adults using Microimplant-assisted Rapid Palatal Expansion (MARPE). Dental Press J Orthod.

[8] Iseri H, Tekkaya AE, Oztan O, Bilgiç S (1998). Biomechanical effects of rapid maxillary expansion on the craniofacial skeleton, studied by the finite element method. Eur J Orthod.

[9] Jafari A, Shetty KS, Kumar M (2003). Study of stress distribution and displacement of various craniofacial structures following application of transverse orthopedic forces--a three-dimensional FEM study. Angle Orthod.

[10] Moon W, Wu KW, MacGinnis M, Sung J, Chu H, Youssef G, Machado A (2015). The efficacy of maxillary protraction protocols with the micro-implant-assisted rapid palatal expander (MARPE) and the novel N2 mini-implant-a finite element study. Prog Orthod.

[11] Peterson J, Wang Q, Dechow PC (2006). Material properties of the dentate maxilla. Anat Rec A Discov Mol Cell Evol Biol.

[12] Ghoneima A, Abdel-Fattah E, Hartsfield J, El-Bedwehi A, Kamel A, Kula K (2011). Effects of rapid maxillary expansion on the cranial and circummaxillary sutures. Am J Orthod Dentofacial Orthop.

[13] Cantarella D, Dominguez-Mompell R, Mallya SM, Moschik C, Pan HC, Miller J (2017). Changes in the midpalatal and pterygopalatine sutures induced by micro-implant-supported skeletal expander, analyzed with a novel 3D method based on CBCT imaging. Prog Orthod.

[14] Timms DJ (1980). A study of basal movement with rapid maxillary expansion. Am J Orthod.

[15] Leonardi R, Cutrera A, Barbato E (2010). Rapid maxillary expansion affects the spheno-occipital synchondrosis in youngsters A study with low-dose computed tomography. Angle Orthod.

[16] Silvestrini-Biavati A, Angiero F, Gambino A, Ugolini A (2013). Do changes in spheno-occipital synchondrosis after rapid maxillary expansion affect the maxillomandibular complex. Eur J Paediatr Dent.

[17] Holberg C, Rudzki-Janson I (2006). Stresses at the cranial base induced by rapid maxillary expansion. Angle Orthod.

[18] Holberg C (2005). Effects of rapid maxillary expansion on the cranial base-an FEM-analysis. J Orofac Orthop.

[19] Wolff J (1892). Das Gesetz der transformation der Knochen.

[20] Wroe S, Ferrara TL, McHenry CR, Curnoe D, Chamoli U (2010). The craniomandibular mechanics of being human. Proc Biol Sci.

[21] Reilly DT, Burstein AH (1975). The elastic and ultimate properties of compact bone tissue. J Biomech.

[22] Verrue V, Dermaut L, Verhegghe B (2001). Three-dimensional finite element modelling of a dog skull for the simulation of initial orthopaedic displacements. Eur J Orthod.

[23] Lee HK, Bayome M, Ahn CS, Kim SH, Kim KB, Mo SS (2014). Stress distribution and displacement by different bone-borne palatal expanders with micro-implants a three-dimensional finite-element analysis. Eur J Orthod.

[24] Melsen B (1969). Time of closure of the spheno-occipital synchondrosis determined on dry skulls A radiographic craniometric study. Acta Odontol Scand.

[25] Lee SP, Paik KS, Kim MK (2001). Anatomical study of the pyramidal process of the palatine bone in relation to implant placement in the posterior maxilla. J Oral Rehabil.

[26] Melsen B, Melsen F (1982). The postnatal development of the palatomaxillary region studied on human autopsy material. Am J Orthod.

[27] Holberg C, Steinhäuser S, Rudzki-Janson I (2007). Rapid maxillary expansion in adults cranial stress reduction depending on the extent of surgery. Eur J Orthod.

[28] Baldawa RS, Bhad WA (2011). Stress distribution analysis during an intermaxillary dysjunction A 3-D FEM study of an adult human skull. Ann Maxillofac Surg.

[29] Thilander B, Ingervall B (1973). The human spheno-occipital synchondrosis II. A histological and microradiographic study of its growth. Acta Odontol Scand.

[30] Boryor A, Geiger M, Hohmann A, Wunderlich A, Sander C, Martin Sander F (2008). Stress distribution and displacement analysis during an intermaxillary disjunction-a three-dimensional FEM study of a human skull. J Biomech.

